# Gallium ions incorporated silk fibroin hydrogel with antibacterial efficacy for promoting healing of *Pseudomonas aeruginosa*-infected wound

**DOI:** 10.3389/fchem.2022.1017548

**Published:** 2022-10-28

**Authors:** Hui Zheng, Zhida Huang, Tongxin Chen, Yafeng Sun, Shouqing Chen, Guangming Bu, Hongcai Guan

**Affiliations:** ^1^ Wenzhou Institute of Industry & Science, Wenzhou, Zhejiang, China; ^2^ Ruibang Laboratories, Wenzhou, Zhejiang, China

**Keywords:** silk fibroin protein, gallium, hydrogels, against bacterial, wound healing

## Abstract

The continual resistance to antibiotics and the generation of a series of bacterial infections has emerged as a global concern, which requires appropriate measures and therapeutics to address such a menace. Herein, we report on Silk fibroin (SF) hydrogel with good biocompatibility and biodegradability fabricated through the crosslinking of the SF of different concentrations with Gallium nitrate (Ga (NO_3_)_3_) against *Pseudomonas aeruginosa*. However, the SF: Ga = 500: 1 (w/w) (SF/Ga) demonstrated a good bactericidal and wound healing effect as a result of the moderate and prolonged release of the Ga^3+^ following the gradual degradation of the hydrogel. The Ga^3+^, known for its innovative nature acted as a crosslinked agent and a therapeutic agent employing the “Trojan horse” strategy to effectively deal with the bacteria. Also, the Ga^3+^, which is positively charged neutralizes the negative potential value of the SF particles to reduce the charge and further induce the β-sheet formation in the protein structure, a characteristic of gelation in SF. The morphology showed a fabricated homogenous structure with greater storage modulus- G^’^ with low loss modulus- G^''^ modulus demonstrating the mechanical performance and the ability of the SF/Ga hydrogel to hold their shape, at the same time allowing for the gradual release of Ga^3+^. A demonstration of biocompatibility, biodegradability, bactericidal effect and wound healing in *in vitro* and *in vivo* present the SF/Ga hydrogel as an appropriate platform for therapeutic and for antibacterial wound dressing.

## Introduction

On the one hand, bacterial infections have been on the rise as a result of the uncertain resistance generated by the bacteria following the administration of antibiotics. A condition that has largely been attributed to the misuse and continual use of these antibiotics. As a consequence of these more than 14 million individuals are affected each year and continue to be affected with a substantial rise in the case of morbidity and mortality (Wang et al., 2017). On the other hand, and to a great degree, skin injuries occurring from diverse conditions make the body vulnerable to this bacterial infection and even dehydration (Wu et al., 2019; Yu et al., 2022). Therefore, the upkeeping of an organism’s first line of defense from invasion by microorganisms and even homeostasis easily becomes compromised (Zhou et al., 2018; He et al., 2020). All these present the need for a suitable cause of action for the treatment of bacterial infections and rapid means of managing wounds.

Employing polymeric hydrogel owing to its soft tissue-like hydrophilic properties has recently gained prominence when it comes to wound-related cases (Kirker et al., 2002; Mogoşanu and Grumezescu, 2014). Therein, the silk fibroin (SF) a natural protein polymer extracted from the cocoons of the silkworm, *Bombyx mori* have also been extensively studied due to their waterproof nature, biodegradability, gaseous permeation, mild inflammatory potential and homeostatic properties (Minoura et al., 1995; Santin et al., 1999; Gil et al., 2013). These properties allow the SF to easily coordinate between bacteria strains and the antibacterial substance they are reinforced with, thus perfect for antibacterial and wound dressing. Additionally, the modification of the physical parameters of SF aqueous solution, pH, conditions for sonication, and dehydration *via* the addition of ethanol or polymer can trigger the transformation of SF molecules from random-coil/α-helix structures to β-sheets, boosting the physical crosslinking (Hu et al., 2013; Bai et al., 2014; Kambe and Yamaoka, 2019). It is worth noting that, the formation of β-sheets triggers the sol-gel transition of silk fibroin through hydrophobic interaction of molecules, which results in the stabilization of hydrogels (Matsumoto et al., 2006; Nultsch and Germershaus, 2017). Though the influence of mechanical forces and pH on the SF has been extensively studied, the role of ions in the SF hydrogels remains poorly understood. Kelly and co-workers reported that some amino acid residues can emerge as β-turns through chelation with copper ions and subsequently form stable β-sheet by hydrogen bonding (Schneider and Kelly, 1995). Also, some studies show that the folding process of the SF may easily be affected by some ions such as Ca^2+^, Zn^2+^, K^+^, and Na^+^ (Zhou et al., 2004; Zong et al., 2004; Ruan et al., 2008; Ruan and Zhou, 2008). These studies illustrated that the suitable ions may have the ability to induce the sol-gel transition of silk fibroin.

What’s more, one crucial nutrient required by the bacteria for the coordination of DNA synthesis, cellular respiration and even eluding of the destructive activity through the generation of ROS is iron (Fe) (Andrews et al., 2003; Hijazi et al., 2018). On the contrary, Ga^3+^ is an anti-infective agent capable of binding to biological complexes containing Fe due to the resemblance in chemical properties, permitting their infiltration into such essential Fe^3+^ binding sites within protein and enzymes. Consequently, this process can alter the Fe content in the bacteria’s antioxidant enzymes disrupting the redox-driven process and making the bacteria susceptible to oxidants (Bernstein, 1998; Lemire et al., 2013; Goss et al., 2018; Wang et al., 2019). Ga (NO_3_)_3_ has been reported to have a highly significant bactericidal effect against *P. aeruginosa* even at as low concentrations and is not reduced under physiological conditions (Kaneko et al., 2007; Minandri et al., 2014; Chitambar, 2016). However, no prior research has been reported on the influence of the Ga^3+^on the SF. In this study, SF: Ga(III) hydrogels were synthesized through the electrostatic adherence of Ga (NO_3_)_3_ into SF with different concentrations to form a new type of antibacterial hydrogel with ideal mechanical properties and for the first time, we report on the effect against the *P. aeruginosa* and for wound healing. The effect of the SF: Ga(III) = 500: 1 (w/w) (SF/Ga) was investigated *in vitro* and *in vivo*. Positively charged Ga^3+^ could “kill two birds with one stone”, in addition to antibacterial action, it cause the aggregation of fibroin molecules into hydrophilic regions, forming a SF hydrogel through ionic crosslinks. The SF/Ga hydrogel exhibited a highly effective antibacterial efficiency capacity in the *in vitro* with good biocompatibility*.* This was confirmed by the *in vivo* experiments where the SF/Ga hydrogel effectively disrupted the *P. aeruginosa via* the slowly and prolonged release of the Ga^3+^ at the same time stepping up with the wound recovery. In all, SF/Ga hydrogel demonstrate great potential for treating bacterial infections and wound dressing.

## Experiment section

### Materials

*Bombyx mori* cocoons were provided by Chongqing University (China). Gallium nitrate hydrate (99.9% metals basis) was purchased from MACKLIN. Sodium carbonate (Na_2_CO_3_), Boric acid, and Sodium chloride (NaCl) were purchased from Sigma. Lithium Bromide salt (LiBr), sodium hydroxide (NaOH) were purchased from Aladdin. Tryptone Broth was purchased from Sangon Biotech. Dialysis bag (MD44, 3.5 K MWCO) was purchased from Solarbio. CCK-8 was purchased from APE×BIO, and DMEM was purchased from Life Technologies Corporation.

### Preparation of silk fibroin solution

*Bombyx mori* cocoons were degummed to remove sericin proteins; silk cocoons were treated in alkaline water baths (0.02 mol/L Na_2_CO_3_) at 100°C for 2 h. Degummed silk was washed several times in deionized water and dried at room temperature to obtain pure SF fibers. The dried SF was dissolved in 9.3 mol/L LiBr solution and stirred for 1 h at 65 °C. After cooling, the solution was taken out into a dialysis bag, placed in an alkaline buffer solution (Boric acid 18.54 g, NaCl 8.775 g dissolved in 1 L of deionized water, pH titration to 9) dialyzed for 1 day to remove the LiBr salt. The solution was dialyzed against deionized water for 3 days and subsequently filtered. At last, pure SF aqueous solution was obtained, and the final concentration was about 5% (w/v), which was stored at 4°C.

### Determination of MIC value of gallium nitrate on *P. aeruginosa*

Using a 96-well plate, the half-dilution method was used to obtain 128 μg/ml to 1 μg/mL Ga (NO_3_)_3_ solution of 50 μL per well, and 50 μL of *P. aeruginosa* bacterial solution with the value of OD_600_ = 0.05 added to each well. The plate was incubated at 37°C in a constant temperature incubator for 24 h. OD_600_ value was measured with a microplate reader (Thermo scientific).

### Preparation of silk fibroin-gallium nitrate hydrogel

The SF: Ga (III) hydrogels were prepared by mixing Ga (NO_3_)_3_ with SF in solution. The SF solution and Ga (NO_3_)_3_ were mixed in different ratios for use, specifically, the SF solution concentration was maintained at 5% by changing the blend volume ratio, and the mass ratios of SF and Ga (NO_3_)_3_ were 5000:1, 2500:1, 1000:1, 500:1 and 250:1, which were ultrasonicated for 1 h after mixing, and the state of gelation observed after standing. Based on the gel formation time and properties, SF: Ga(III) = 500: 1 (w/w) is expected to be the best ratio.

### SEM and EDS characterizations

The scanning electron microscope (SEM; HITACHI SU8010, Japan) was used to analyze the dried gel morphology. The SF hydrogel and SF/Ga hydrogel were quickly frozen in liquid nitrogen for 10 min, and after, placed in a vacuum freeze dryer (BIOCOOL, China) and freeze-dried at −50°C for 72 h. The sample was pasted on the round sample stage with a conductive double-sided adhesive. Before the test, the sample was processed with gold spray. The thickness of the gold spray was between 20 and 30 nm, and the test was conducted at a temperature of 20°C and relative humidity of 65%.

### CD and FTIR characterizations

CD spectra of samples were measured on an Applied Photophysics (Chirascan Plus, England) spectrometer under an N_2_ atmosphere. Approximately 20 μL of the samples were evenly placed on a 1 mm thick quartz cuvette and scanned from 180 to 260 nm. Three groups of samples (SF solution, SF solution after 1 h of ultrasound, SF solution, and Ga (NO_3_)_3_ were mixed at a mass ratio of 500:1. The samples for the Fourier transform infrared spectrometer (FTIR, Bruker Tensor II, Germany) were also freeze-dried. The wave number range was 2000–1000 cm^−1^, the spectral resolution was 4 cm^−1^, and the scanning range was 4000–400 cm^−1^.

### Rheological test

The mechanical and rheological analyses were carried out using (TA Instrument, New Castle, DE). The elasticity (storage modulus- G’) and viscosity (loss modulus- G'') were evaluated at a shear frequency of 1 Hz within a linear viscoelastic region. All frequency sweeps were collected within a frequency range of 0.1–10 Hz rad/s at 25°C. All samples were stabilized and measured, and the process was repeated three times.

### Zeta potential

The samples [Mixed Ga (NO_3_)_3_ with SF solution (0.05% wt) at 1000:1, 500:1 (w/w), and SF solution only] were characterized with a nanometer particle size analyzer (Malvern, Zetasizer Nano ZS ZEN3600, England) to determine the change of Zeta potential, and each sample was measured three times repeatedly.

### Cell culture and cytotoxicity assay

Human lung fibroblasts (MRC-5) cells were cultured in DMEM medium supplemented with 100 U ml^−1^ penicillin, 0.1 mg ml^−1^ streptomycin, and 10% FBS at 37°C in a humidified incubator under 5% CO_2_. After reaching 80% confluence, cells were harvested and seeded in 96-well plates (2 × 10^4^ cells/well). At the same time, 1 ml PBS solution, SF, and SF/Ga hydrogel were separately put into the 6-well plate, sterilized under UV for 30 min and 5 ml DMEM, was added to each well for co-cultivation. After 24 h, the culture medium was replaced with hydrogel leaching solution for each group and cells were cultured for 1 day. The cytotoxicity was measured by the CCK-8 method.

### Antibacterial property

The antibacterial activities of the SF/Ga hydrogel against *P. aeruginosa* bacteria were evaluated using the oscillation method. LB culture medium was used to dilute *P. aeruginosa* solution which was recovered for 24 h, OD_600_ = 0.05. The bacterial solutions (4 ml) were added to 15 ml shake tubes and later divided into 3 groups: 1 ml of SF hydrogel, SF/Ga hydrogel, and Ga (NO_3_)_3_ solution. The samples were cultured in a 37°C constant temperature incubator, and Shaked for 7 days. The samples (100 μL) were taken out every day and the OD_600_ was measured.

### Wound healing

To evaluate the potential of the SF/Ga hydrogel for wound dressing, an *in vivo* experiment was conducted using a rat model with a *P. aeruginosa*-infected wound. (He et al., 2022a; He et al., 2022b). Six 3-month-old healthy Sprague–Dawley (SD) rats were randomly divided into 2 groups (PBS and SF/Ga hydrogel treatments) and anesthetized with chloral hydrate. The back fur of the mice was shaved, cleaned, and disinfected with 75% alcohol and an 8-mm biopsy punch was used to create circular wounds of full thickness, 8 mm in diameter on each rat. The wounds were infected with 100 μL of 10^8^ CFU/mL *P. aeruginosa* solution. Images of the wound area at 0, 1, 2, 3, and 6 days were taken with a digital camera, and the percentage of wound size was calculated using ImageJ software.

### Statistical analysis

All the experiments were performed with sample sizes greater than three, and the experimental results were expressed as the mean value ±standard deviation. The difference between the groups was determined using a one-way analysis of variance, and a *p*-value smaller than 0.05 was considered a statistically significant difference.

## Result and discussion

### Susceptibility of *P. aeruginosa* to Ga (NO_3_)_3_

**FIGURE 1:**
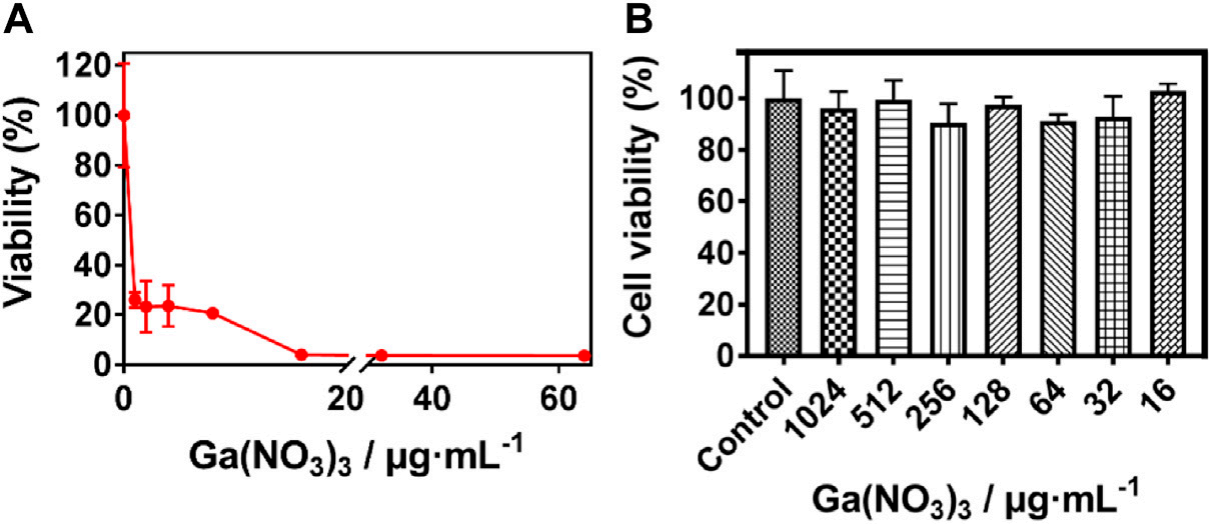
Susceptibility of *P. aeruginosa* to Ga (NO_3_)_3_. **(A)** Minimum inhibition concentration of Ga (NO_3_)_3_ against *P. aeruginosa*, **(B)** Cytotoxicity of Ga (NO_3_)_3_ to MRC-5 (mean ± SD, *n* = 3).

The release of Ga^3+^ is not able to appropriately replace the Fe^3+^ within the protein binding site of bacteria since the Ga^3+^ transition to Ga^+^ requires much energy compared to the transition of Fe electrons from 3 + to 2 + or *vice versa* (Ma et al., 2013; Nikolova et al., 2016; Best et al., 2020). Hence the disruption of the crucial processes by Ga^3+^ after replacing the Fe^3+^ and the subsequent antibacterial action. The susceptibility of *P. aeruginosa* was investigated to screen the concentration of Ga (NO_3_)_3_ used in the SF: Ga (III) hydrogel fabrication. As shown in Figure 1A, the Minimum inhibition concentration (MIC) of Ga (NO_3_)_3_ was recorded as 16 μg/ml which was sufficient to completely disrupt the *P. aeruginosa*. Also, a measure of the toxicity of the different concentrations of Ga (NO_3_)_3_ on the human lung fibroblast (MRC) using the MTT assay, Figure 1B showed that an increment in the concentration of Ga (NO_3_)_3_ from 16 μg/ml to 1024 μg/ml yielded cell viability which was above 80%. This indicated the negligible toxicity of the concentration of Ga (NO_3_)_3_ within this interval.

### Preparation of the SF: Ga (III) hydrogels

**FIGURE 2:**
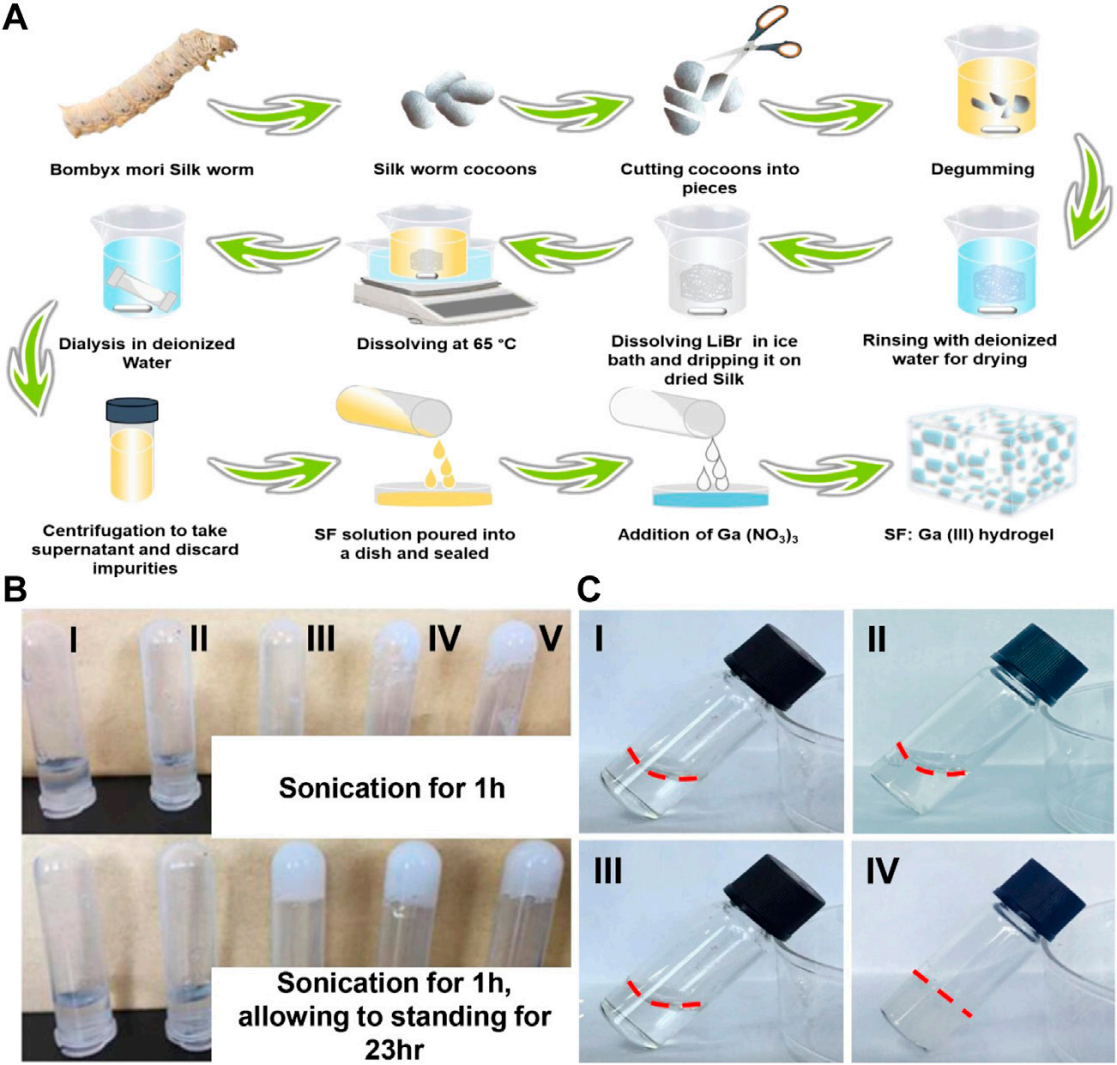
Synthesis of hydrogels. **(A)** Schematic illustration of the preparation of SF and SF: Ga (III) hydrogels, **(B)** Digital images of (I) SF: Ga (III) = 5000: 1 (w/w), (II) SF: Ga (III) = 2500: 1 (w/w), (III) SF: Ga (III) = 1000: 1 (w/w), (IV) SF: Ga (III) = 500: 1 (w/w) (SF/Ga) and (V) SF: Ga (III) = 250: 1 (w/w) hydrogels, **(C)** Digital image of SF solution (I, II) and SF: Ga (III) = 500: 1 (w/w) (SF/Ga) hydrogel (III, IV) before and after sonication and standing for 23 h.

The SF: Ga (III) hydrogels were successfully fabricated after sonication for 1 h and standing for 23 h to form a hydrogel with increased stability, the presence of amino and carboxyl groups allowed for the easy chemical modification. Thus, the addition of Ga (NO_3_)_3_ solution of varying concentrations (12.5 µg, 25 μg, 50 μg, 100 μg, and 200 µg) and the subsequent induction of the gelation of the mixed solution *via* sonication, Figure 2A. The even dispersion of Ga (NO_3_)_3_ within the SF formed a hydrogel network SF: Ga (III) resulting from the self-crosslinking. As shown in, Figure 2B, the uniform dispersion allowed SF: Ga (III) to release bioactive Ga^3+^ at the same time providing a moist environment and enough mechanical support while holding their shapes. The antibacterial effect and the gelation time led to the selection of the 100 μg/ml, Ga (NO_3_)_3_, combined with the SF hydrogel to give SF: Ga (III) = 500: 1 (w/w) (SF/Ga), Figure 2C. The time for the gelation is dependent on the concentration of the gelatine and gel strength also significantly increases with an increase in the gelation concentration. Considering the varying ratio of SF: Ga (III), gelation was expected to decrease from the highest concentration (5000–250) to the lowest with time. However, on average the hydrogels demonstrated a shorter gelation time. Hence, a positive correlation in SF/Ga with their crosslinking and other physical properties.

### Morphological analysis of the SF/Ga hydrogel

**FIGURE 3:**
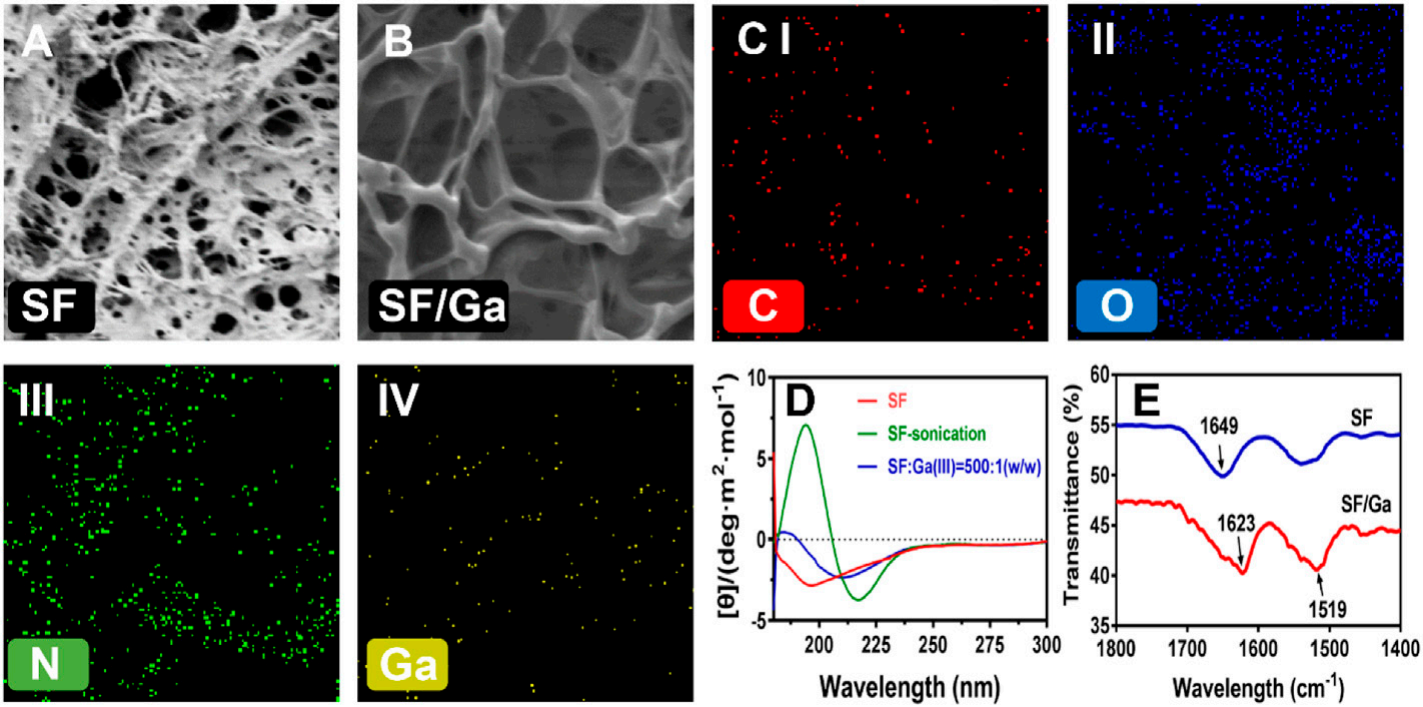
Morphological and secondary structural characterization. **(A,B)** SEM images of the surface pore of SF and SF/Ga hydrogel, **(C)** Elemental mapping of SF/Ga hydrogel, **(D)** CD spectrum of unsonicated SF, ultrasonicated SF, and sonicated SF/Ga solutions, **(E)** FTIR of SF and SF/Ga hydrogel.

A high proportion of the SF is bound to increase the viscosity of the hydrogel solution, inducing a short gelation time which can restrict the crosslinking and at the same time affect the porosity (Xu et al., 2021). The SEM evaluation of the influence of Ga (NO_3_)_3_ on the micromorphological structure of the hydrogel after dried freezing revealed a lamellar interconnected pores size for SF hydrogel, Figure 3A, and a smaller but increased pore size for Ga (NO_3_)_3_ crosslinked hydrogel SF/Ga, Figure 3B. In all, the morphological evaluation shows the hydrogel was three-dimensionally porous. Elemental analysis of SF/Ga hydrogels with an X-ray energy spectrometer (EDS) attached to the high-resolution electron microscope, confirmed the presence of C, N, O, and Ga elements in the fabricated SF/Ga hydrogel, where C, N, and O are components of silk fibroin and, Ga a mixed component of Ga (NO_3_)_3_, Figure 3C. The changes in the protein secondary structure of the SF/Ga were explored using circular dichroism (CD) and compared with those of unsonicated SF solution and ultrasonicated SF within 180–260 nm. As shown in Figure 3D, CD spectrum of the unsonicated SF solutions revealed a negative intensity peak close to 195 nm, this indicates a typical random coil structure. In addition, the CD spectrum of the sonicated SF solution showed a negative and positive intensity peak at 217 nm and 198 nm respectively, which is more typical of a characteristic peak within β-sheet conformation. Interestingly addition of Ga (NO_3_)_3_ to the SF demonstrated a change that accompanied the SF/Ga switching from a random coil structure to a β-sheet conformation which was consistent with the change in the conformation of the SF after adding Ga (NO_3_)_3_. Emphasizing that the β-sheet is the dominant secondary structure in the SF/Ga. Figure 3E shows the FTIR spectra which investigated the interaction between Ga (NO_3_)_3_ and the SF, and this reveals a spectra peak of amide I (1700–1600 cm^−1^) and amide II (1600–1500 cm^−1^) regions for the SF. The SF conformation comprises a random coil, α-helix, β-sheet, and β-turn. A characteristic peak with the interval of 1625–1640 cm^−1^ (amide I) and 1515–1525 cm^−1^ (amide II) indicated that the secondary structure is related to the β-sheet. Similarly, the peaks of 1640–1660 cm^−1^ (amide I) and 1535–1550 cm^−1^ (amide II) indicated that the secondary structure of the protein is dominated by the α-helix and random coil. However, peaks identified at 1700–1600 cm^−1^ (amid I) and amide II (1600–1500 cm^−1^) of SF represented relative maturity. Figure 3E, also showed that 1649 cm^−1^, 1625 cm^−1^, and 1519 cm^−1^ are characteristic absorption peaks of SF amide I, amide I, and amide II, respectively, indicating that pure silk fibroin has a random coil structure. But the addition of Ga (NO_3_)_3_ to the SF, shifted the position of the characteristic peak of the SF to the right, that is, it changed to the silk II conformation, showing an antiparallel β-sheet. Hence, the β-sheet structure is a significant characteristic of SF, giving SF a good mechanical property. This also shows the successful incorporation of Ga (NO_3_)_3_ in the SF/Ga hydrogel.

### Mechanical and rheological properties of SF/Ga

**FIGURE 4:**
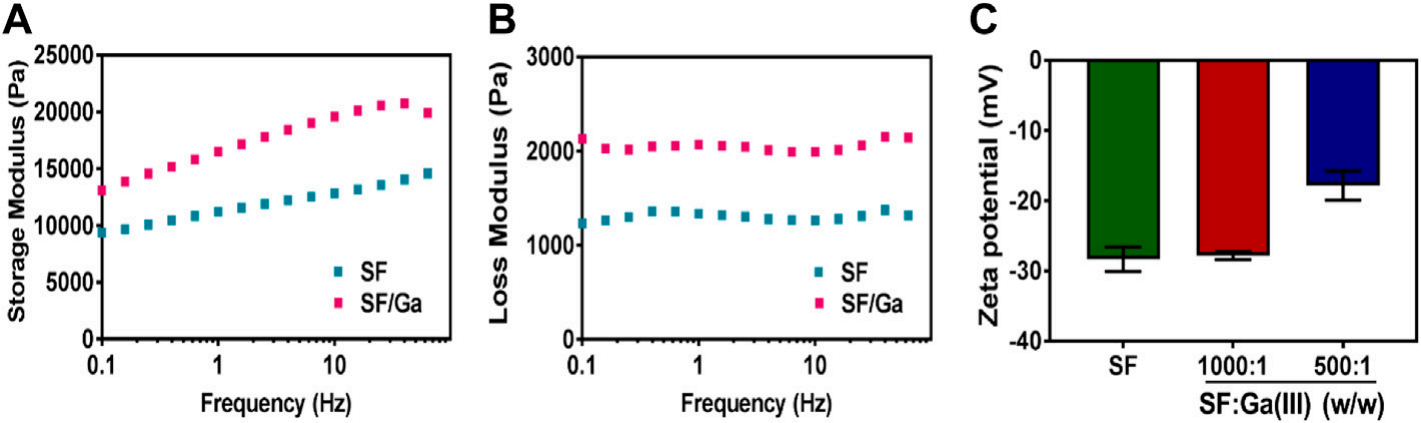
**(A,B)**Rheological characterization and **(C)** Zeta-potentials **(B)** of SF and SF/Ga hydrogel (mean ± SD, *n* = 3).

Some reports indicate that the β-sheet is directly responsible for a silk hydrogel mechanical property as a result of improved crosslinks (Hu et al., 2010; Guziewicz et al., 2011; Xu et al., 2021). Hence crosslinking coordinates and improves the mechanical performance of a hydrogel further enabling resistance to easy deformation (Rammensee et al., 2006; Zawko et al., 2009; Jeon et al., 2014). The influence of Ga (NO_3_)_3_ on the SF hydrogels and the viscosity difference were evaluated *via* a dynamic oscillatory rheological measurement of the storage modulus- G^’^ and loss modulus- G^''^ of sample SF and SF/Ga against time and frequency. Mechanical spectra of SF and SF/Ga showed that the storage modulus- G’ highly exceeded the loss modulus- G''. An indication of a well-obtained hydrogel network, Figures 4A,B. However, a time sweep at a frequency of 1 Hz showed that the SF/Ga had significant stability compared to the SF which could be attributed to Ga (NO_3_)_3_ concentration and the presence of the β-sheet conformation from the cross-linking. The Zeta potential is the magnitude of change that determines the stability of a colloidal suspension. As shown in Figure 4C, the SF revealed a negative Zeta potential value of about −30 mV. However, an increase in the content of Ga (NO_3_)_3_ in the hydrogel significantly decreases the value of the Zeta potential. An indication of possible electrostatic adsorption between the positively charged Ga^3+^, of Ga (NO_3_)_3,_ and the negatively charged SF. Also, this demonstrated that the configuration of the SF and SF: Ga (III) = 1000: 1 (w/w) were random coils, whereas the neutralization process by the positively charged Ga^3+^ yielded a β-sheet in the SF/Ga.

### *In vitro* antibacterial activity and biocompatibility

**FIGURE 5:**
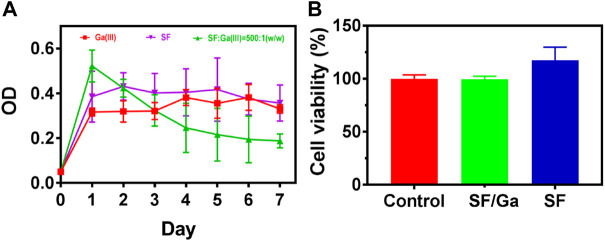
*In vitro* antibacterial activity **(A)** and biocompatibility **(B)** of SF/Ga hydrogel (mean ± SD, n = 3).

Gallium compounds have been employed in many areas in the biomedical field for the treatment of many infectious diseases and even in cancer treatment (Rudnev et al., 2006; Rzhepishevska et al., 2011; Deliormanlı, 2015). Thus, we investigated the antibacterial efficiency of the SF/Ga against *P. aeruginosa*. The effect of 100 μg/mL Ga (NO_3_)_3_ solution, 50 mg/ml pure SF solution, and SF/Ga on the growth of *P. aeruginosa* showed a lower OD value for Ga (NO_3_)_3_ group compared to the SF/Ga group and a high OD value for the SF group, Figure 5A. The significant antibacterial activity observed in Ga (NO_3_)_3_ could be attributed to the release of Ga^3+^ while the high OD value in the SF hydrogel group, indicated the insignificant antibacterial effect. Results from the second day showed the gradual degradation of the hydrogel in the SF/Ga group with a gradual decline in the OD value. The gradual decline in the OD value demonstrated the gradual release of Ga^3+^ as the hydrogel gradually degrades. Extension of time to the 7th day demonstrated a significantly reduced OD value in the SF/Ga group compared to the other two groups. Intriguingly a critical observation of the OD value showed that the bacteria suspension in the Ga (NO_3_)_3_ group began to rise steadily from the 3rd day. This can be ascribed to the hydrolysis of Ga (NO_3_)_3_ which reduces the activity of Ga (NO_3_)_3_ and permitted the bacteria regrowth. This also proves the SF/Ga hydrogel can present a prolonged and efficient antibacterial effect. Also, apart from the antibacterial activity, biocompatibility serves as a direct prerequisite for a biomedical purposes. The *in vitro* cytocompatibility was examined using human lung fibroblast (MRC -5) cells in a CCK-8 assay. As shown in Figure 5B, cells inoculated with SF and SF/Ga hydrogels leaching solution for 24 h demonstrated a cell activity of about 100% relative to the control group, indicating negligible cytotoxicity. However, cells in the SF hydrogels group demonstrated an obvious cell proliferation effect. SF/Ga hydrogel proves good biocompatibility with an efficient antibacterial effect.

### *In vivo* antibacterial activity

**FIGURE 6:**
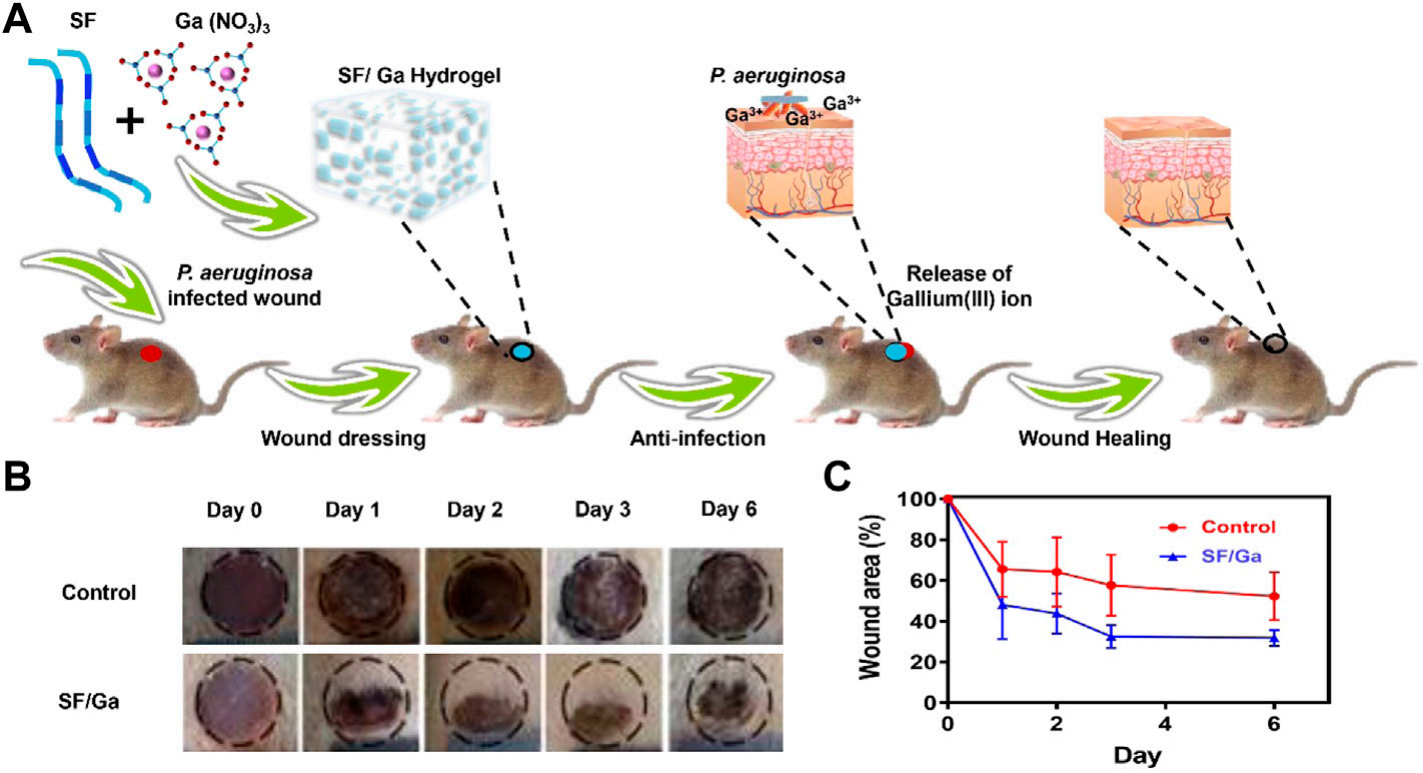
*In vivo* antibacterial activity of SF/Ga hydrogel. **(A)** Schematic illustration of SF/Ga hydrogel promotion of healing of a *P aeruginosa*-infected wound, **(B)** Digital images of *P. aeruginosa* infected wound covered with PBS, and SF/Ga hydrogel **(C)** Quantitative curve of wound area for treatment groups, PBS and SF/Ga hydrogel (mean ± SD, n = 6).

The result from the *in vitro* antibacterial activity and biocompatibility inspired us to further investigate the *in vivo* antibacterial activity of SF/Ga hydrogel. A *P. aeruginosa* infection model in the SD rats was created by making skin incisions on the back of the rats, PBS solution, and SF/Ga hydrogel were applied separately, Figure 6A. The wound recovery in the control group was very slow. In contrast, the SF/Ga group demonstrated a better and more pronounced wound recovery effect Figure 6B. Images of wounds taken at a separate time interval from 0,1,2,3 to 6 days showed that the wound treated with SF/Ga hydrogel exhibited a wound area of about 70% relative to the control group on the 3rd day, an indication of the release of Ga^3+^ for bactericidal effect in the SF/Ga group and with time the wound size began to decrease Figure 6C. On the 6th day, the wound scar rate and closure of the wound in the SF/Ga group were observed to be better compared to the other group, indicating the SF/Ga hydrogel which contains Ga (NO_3_)_3_ wielded the potential to effectively disrupt bacterial activity and at the same time accelerating the wound healing process. This confirms results from the *in vitro* antibacterial activity and presents SF/Ga as an appropriate biomaterial for antibacterial effects and wound healing.

## Conclusion

In summary, we successfully prepared SF/Ga hydrogel and systematically investigated the therapeutic and wound healing ability against *P. aeruginosa*. The SF/Ga hydrogel showed a good water content, biocompatibility, mechanical strength, and biodegradability. The hydrogel allowed the gradual and prolonged release of Ga^3+^ which in turn gradually disrupted the bacteria. Besides SF/Ga hydrogel demonstrated negligible toxicity towards human lung fibroblast (MRC-5) cells affirming their biocompatibility. More importantly, the SF/Ga hydrogel effect on *P. aeruginosa* infected wound exhibited effective recovery confirming its ability to disrupt bacteria and at the same time induce wound healing. Therefore SF/Ga hydrogel can serve as an excellent multifunctional candidate for wound dressing with a prolonged antibacterial effect.

## Data Availability

The original contributions presented in the study are included in the article/supplementary materials, further inquiries can be directed to the corresponding author.
